# Statin treatment after acute coronary syndrome: Adherence and reasons for non-adherence in a randomized controlled intervention trial

**DOI:** 10.1038/s41598-019-48540-3

**Published:** 2019-08-19

**Authors:** Huber Daniel, Wikén Christian, Henriksson Robin, Söderström Lars, Mooe Thomas

**Affiliations:** 1 Department of Public Health and Clinical Medicine, Unit of Research, Education and Development - Östersund, Umeå University, Östersund, Sweden; 2 Unit of Research, Development and Education, Region Jämtland Härjedalen, Östersund Hospital, Östersund, Sweden

**Keywords:** Cardiology, Myocardial infarction, Public health

## Abstract

Studies of secondary prevention for cardiovascular disease show low fulfilment of guideline-recommended targets. This study explored whether nurse-led follow-up could increase adherence to statins over time and reasons for discontinuation. All patients admitted for acute coronary syndrome at Östersund hospital between 2010–2014 were screened for the randomized controlled NAILED-ACS trial. The trial comprises two groups, one with nurse-led annual follow-up and medical titration by telephone to reach set intervention targets and one with usual care. All discontinuations of statins were recorded prospectively for at least 36 months and categorized as avoidable or unavoidable. Kaplan-Meier estimates were conducted for first and permanent discontinuations. Predictors for discontinuation were analysed using multivariate Cox regression, statin type and mean LDL-C at end of follow-up. Female gender was a predictor for discontinuation. Allocation in the intervention group predicted increased risk for a first but decreased risk for permanent discontinuation. A nurse-led telemedical secondary prevention programme in a relatively unselected ACS cohort leads to increased adherence to statins over time, greater percentage on potent treatment and lower LDL-C compared to usual care. An initially increased tendency toward early discontinuation in the intervention group stresses the importance of a longer duration of structured follow-up.

## Introduction

Statin use in secondary prevention after acute coronary syndrome (ACS) is one of the most researched areas in cardiovascular medicine. The ability of statins to reduce low-density lipoprotein cholesterol (LDL-C) as one of the most important factors in the atherosclerotic process has been proven in a number of large-scale randomized trials^1–3^. Although the information is debated, statins have also been proven safe and tolerable^4^. Despite this, epidemiological studies suggest that a large proportion of patients that would benefit from treatment do not reach adequate doses after discharge^5^. Statins are thought to be affected by negative expectations about side effects and effect of treatment, the nocebo effect. These psychological beliefs are influenced by the perceptions of patients, physicians and the media, and may influence long-term adherence^6^. Earlier prospective studies on adherence to statins in secondary prevention generally concentrate on the first 12 months because that time frame is considered the most vulnerable, even though the increased risk for repeated events continues to persist. There is a lack of data from long-term, prospective, randomized, controlled adherence studies in fairly unselected populations as wells as data concerning the exact reasons for treatment discontinuation.

This study aimed to prospectively measure long-term adherence to statins in the Nurse-based Age-independent Intervention to Limit Evolution of Disease after Acute Coronary Syndrome (NAILED ACS) trial and assess whether the intervention improved adherence compared with usual care. Second, we aimed to quantify termination of statin intake and register exact reasons for termination. We also tried to identify predictors of non-adherence.

## Results

**Figure 1:**
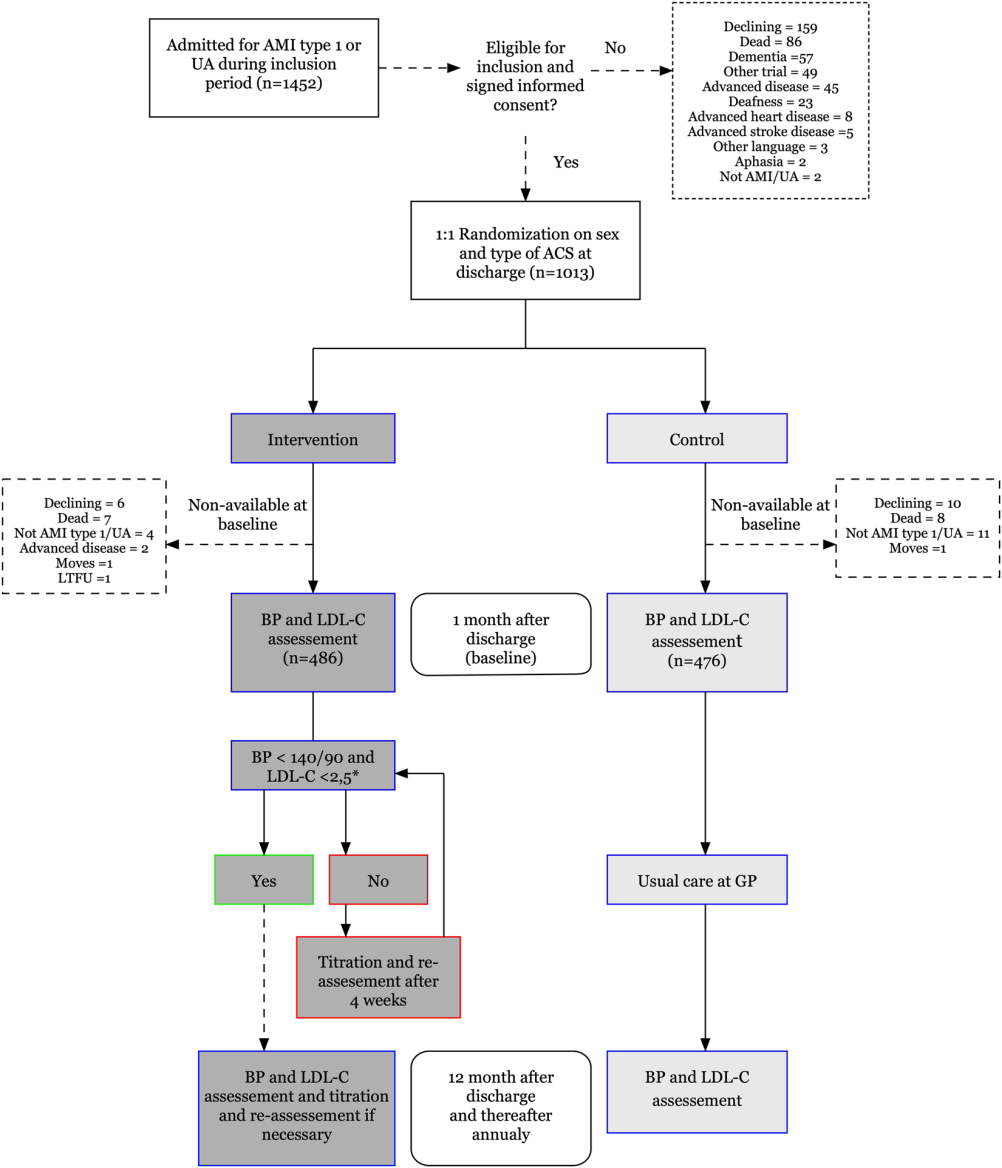
Flow chart of study participants.

During the set inclusion time, 1013 patients were alive and included at discharge. At baseline follow-up, 22 patients in the intervention group did not participate (8 dead, 4 unwilling to participate, 4 excluded for advanced disease, 4 not type-1 AMI/UA, 2 lost to follow-up) and 29 in the control group (9 dead, 9 unwilling to continue, 11 not type-1 AMI/UA) (Fig. 1).

There were no significant differences in baseline characteristics (Table 1).

**Table 1:** Baseline characteristics.

*Patient characteristics*	Control (N = 476)	Intervention (N = 486)	p
Age (mean)	69	68	0.091
Women, n (%)	144 (30)	146 (30)	0.601
BMI kg/m^2^ (mean)	27.4	27.8	0.346
Current smoker, n (%)	90 (18.9)	97 (20)	0.53
Former smoker^a^, n (%)	196 (41.2)	211 (43.5)	0.53
Heredity^b^, n (%)	129 (27.4)	136 (28.5)	0.56
Basic education, n (%)	241 (51.0)	224 (46.3)	0.32
Cholesterol mmol/L, mean (SD)	4.1 (1)	4.1 (0.91)	0.848
Ldl-C mmol/L, mean (SD)	2.18 (0.85)	2.18 (0.74)	0.961
***ACS diagnosis, n (%)***			
STEMI	131 (27.5)	152 (31.3)	0.201
NSTEMI	296 (62.2)	295 (60.7)	0.636
UA	49 (10.3)	39 (8.0)	0.222
***Revascularization, n (%)***			
PCI	249 (52.3)	261 (53.7)	0.665
CABG	59 (12.4)	65 (13.4)	0.650
***Comorbidities, n (%)***			
History of AMI	96 (20.2)	79 (16.3)	0.116
History of PCI/CABG	89 (18.6)	69 (14.2)	0.210
History of ischaemic stroke/TIA	30 (7.3)	27 (5.6)	0.949
History of hypertension	267 (56.1)	263 (54.1)	0.538
History of diabetes	96 (20.2)	99 (20.4)	0.938
History of hyperlipidemia	335 (70.4)	335 (68.9)	0.521
AF	40 (8.4)	44 (9.0)	0.578
***Baseline medication, n (%)***			
Lipid lowering treatment^c^	442 (92.8)	451 (92.7)	0.827
Beta-blocker	429 (90.3)	440 (90.9)	0.753
ACE inhibitor/ARB	382 (80.2)	395 (81.3)	0.350
Aspirin	446 (93.9)	446 (92.1)	0.289
P2Y12-inhibitor^d^	372 (78.1)	375 (77.2)	0.885
Anticoagulant^e^	33 (6.9)	44 (9.0)	0.346

^a^Former smoker: smoking cessation > 6 months; ^b^Heredity: first-degree relative with cardiovascular disease, male <55, female <65; ^c^Statin or ezetimibe; ^d^P2Y12 inhibitors: clopidogrel, ticagrelor, prasugrel; ^e^Anticoagulant: warfarin, non-vitamin K oral anticoagulant. BMI – Body mass index; STEMI – ST elevation myocardial infarction; NSTEMI – Non-ST elevation myocardial infarction; UA – Unstable angina; PCI – Percutaneous coronary intervention; CABG – Coronary artery bypass graft; AMI – Acute myocardial infarction; TIA – Transitory ischaemic attack.

### Adherence

**Figure 2:**
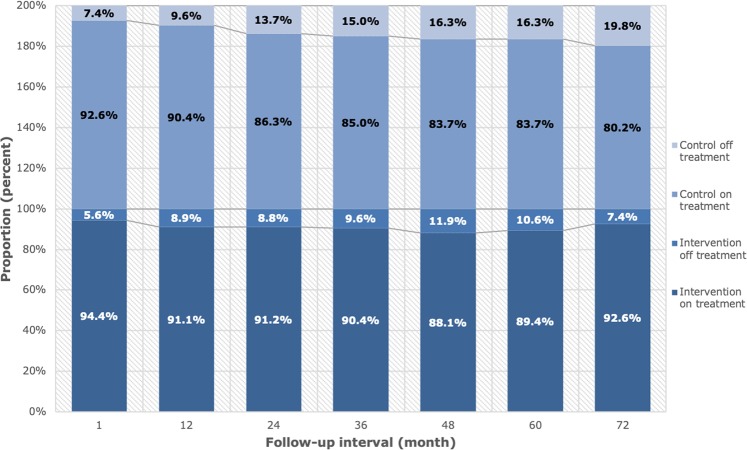
Adherence at each assessment interval.

Of the remaining 962 patients, 89.5% (n = 435) in the intervention and 85.2% (n = 390) in the control group were adherent to statin treatment after a mean of 3.9 years of follow-up (p < 0.05). A total of 27.8% (n = 135) of patients in the intervention group and 20.8% (n = 99) of patients in the control group discontinued statin treatment at any time during the period (p < 0.05). The percentages of patients on treatment at each scheduled follow-up are presented in Fig. 2.

**Figure 3:**
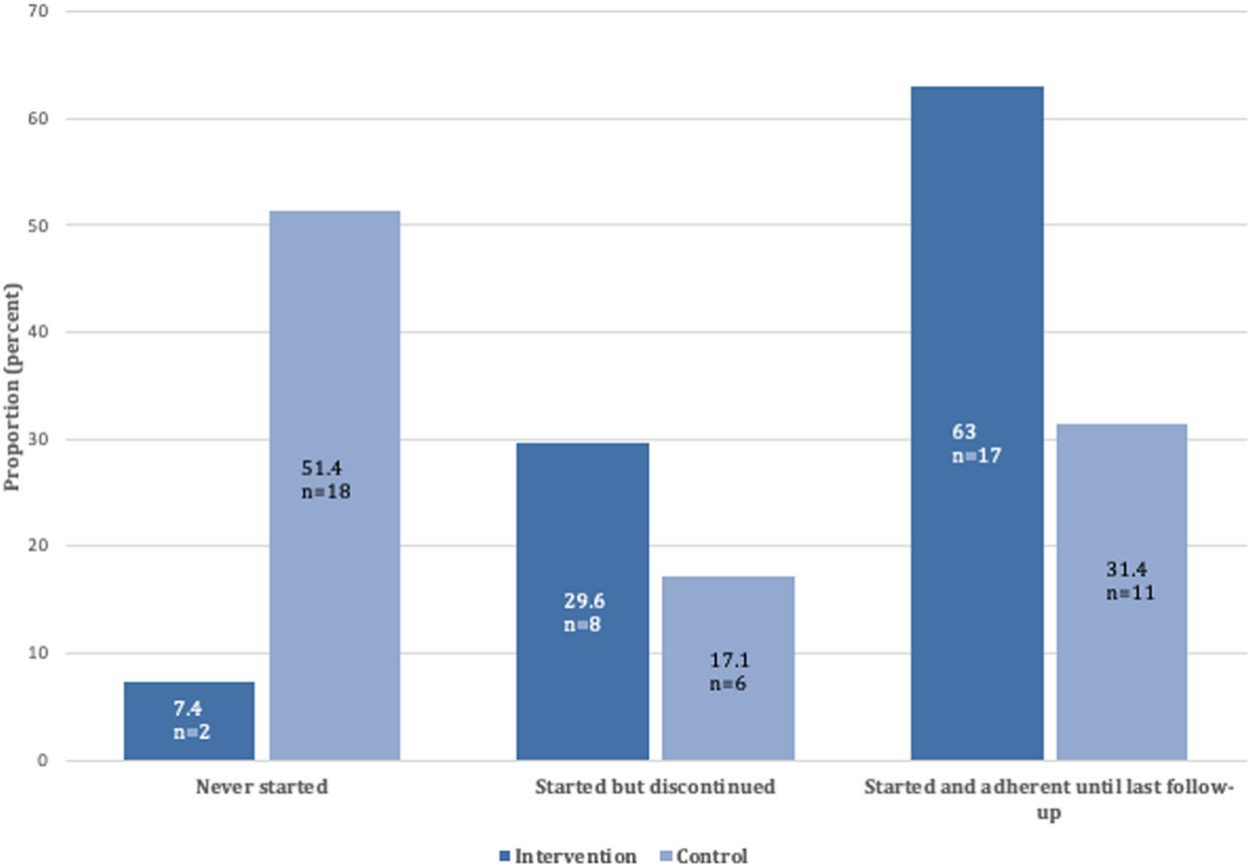
Outcomes of patients not initiated on statins at discharge.

Of those who discontinued at any time, the mean number of discontinuations during the study period was 1.31 (SD 0.60) in the intervention group and 1.15 (SD 0.39) in the control group (p < 0.05). In an attempt to further assess the effect of the intervention, we analysed the outcome of those not on statin treatment at discharge (intervention n = 27, control n = 35). Of 27 patients in the intervention group, 92.6% (n = 25) were initiated on statin treatment and 67% (n = 18) persisted on treatment at last follow-up; of 35 patients in the control group, 45.7% (n = 16) initiated treatment and 28.6% (n = 10) persisted, p < 0.001 (Fig. 3).

### Reasons for discontinuation

**Figure 4:**
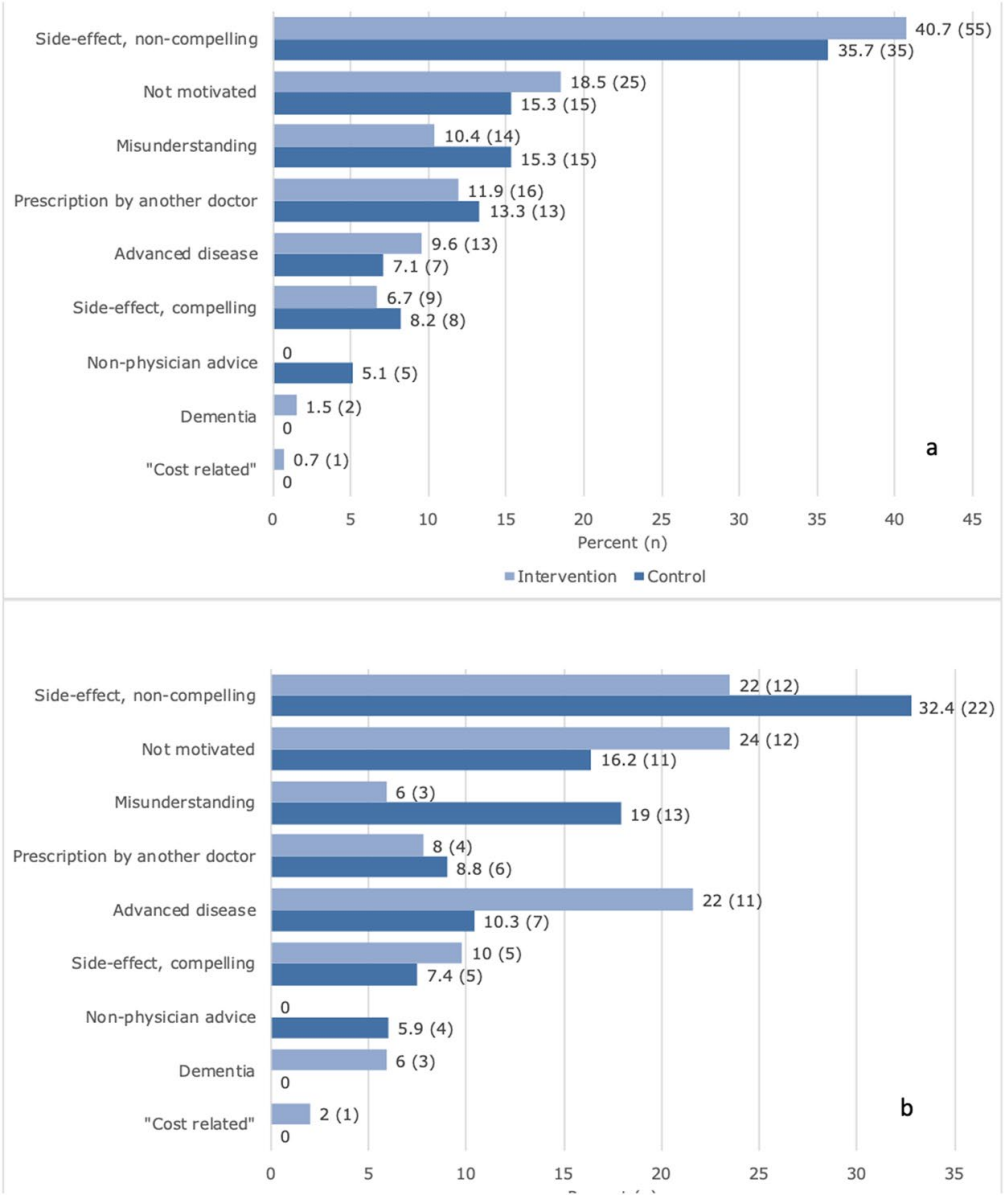
Reasons for discontinuation. (**a**) first discontinuation; (**b**) permanent discontinuation. Presented group-wise. Patients that never initialised statin treatment are excluded.

The most prevalent reason for permanent discontinuation in the intervention group was advanced disease including dementia (27.5%, n = 14); in the control group, it was side effects without a compelling relation to treatment (32.4%, n = 22) (Fig. 4). In both study arms, the most common reasons for a first discontinuation were side effects without a compelling relation to treatment and lack of treatment motivation, p = 0.15 (Fig. 4).

**Figure 5:**
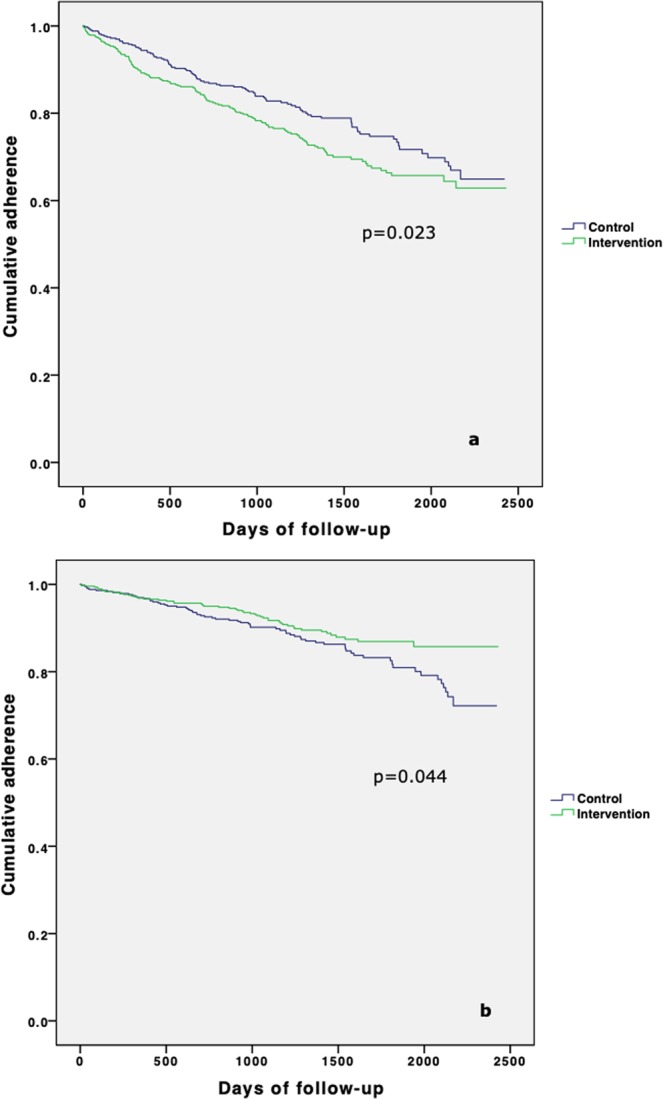
Kaplan-Meier plots on first (**a**) and permanent (**b**) discontinuation.

Kaplan-Meier estimations for a first and permanent discontinuation are presented in Fig. 5.

### Predictors of discontinuation

In the Cox regression of factors associated with a first discontinuation, participation in the intervention group was associated with an increased risk and STEMI as inclusion event with a decreased risk. In the second Cox regression model of predictors for permanent discontinuation, both STEMI and participation in the intervention group led to decreased risk. After adjusting for non-proportional hazard, female gender was significantly associated with both first and permanent discontinuation, with a linearly decreasing risk over time until the risk was equalized (1250 days for first discontinuation and 900 days for permanent) (Table 2).

**Table 2:** Predictors for a first and permanent discontinuation.

	Factor	Hazard Ratio (Standard Error)
Predictors for a first discontinuation	Randomization to the intervention group	1.35 (0.13)
	Female gender	1.23 (0.14)*
	STEMI as including event	0.74 (0.15)
Predictors for a permanent discontinuation	Female gender	1.45 (0.19)*
	Randomization to the intervention group	0.69 (0.19)
	STEMI as including event	0.63 (0.22)

Non-proportional hazard for female gender. *Mean hazard ratio during the studied period. p < 0.05 for all predictors.

### Intensity of treatment

Mean LDL-C at last recorded assessment was 2.00 (SD 0.92) in the intervention group and 2.39 (SD 1.03) in the control group (p < 0.001). LDL-C at last follow-up was not recorded for 6 patients in the intervention group and 5 in the control group due to hypertriglyceridemia where LDL-C could not be calculated. At the end of follow-up, 337 (69.3%) in the intervention group were treated with a high-intensity LDL-lowering regimen, compared with 211 (44.3%) in the control group (p < 0.001). In addition, 104 patients (21.4%) in the intervention group were also treated with ezetimibe (n = 103) or gemfibrozil (n = 1) and 10 (1.2%) in the control group were treated with ezetimibe (p < 0.001). Types of lipid lowering therapy are presented in Table 3.

**Table 3:** Type of lipid-lowering treatment at end of follow-up.

	Primary lipid lowering therapy		Additional therapy	
	Intervention	Control	Intervention	Control
None, n (%)	49 (10.1)	84 (17.6)	382 (78.6)	466 (97.9)
Simvastatin, n (%)	56 (11.5)	110 (23.1)		
Atorvastatin, n (%)	298 (61.3)	256 (53.8)		
Rosuvastatin, n (%)	72 (14.8)	19 (4.0)		
Other statin, n (%)	5 (1)	3 (0.6)		
Ezetimibe, n (%)	6 (1.2)	3 (0.6)	104 (21.4)	10 (1.2)
Other, n (%)	0	1 (0.2)	1 (0.2)	0
High-intensity, n (%)	337 (69.3)	211 (44.3)		
Low/Medium-intensity, n (%)	94 (19.3)	177 (37.2)		

High-intensity: lipid-lowering therapy (simvastatin >40 mg, atorvastatin ≥40 mg or rosuvastatin >10 mg); Low/Medium-intensity: not fulfilling the above.

## Discussion

In this study, the rate of adherence to statin treatment was almost 90% after a mean follow-up time of 3.8 years in a community-based ACS population with a nurse-led telephone-based intervention. The rate was significantly higher than in the usual care control group (85%). The most prevalent reasons for permanent discontinuation in the intervention group were non-avoidable causes (advanced disease including dementia), whereas in the control group the most common reasons were avoidable (non-compelling side effects and misunderstanding). In both groups, the most prevalent reasons for a first discontinuation were avoidable (side effects without a compelling relation to treatment, lack of motivation and misunderstanding). The intervention group was more often treated with high-intensity LDL-lowering medication and had significantly lower mean LDL-C than controls at last follow-up. Female sex predicted non-adherence and STEMI predicted increased adherence. Patients in the intervention group had a higher rate of a first discontinuation but a lower rate of a permanent discontinuation.

There is an inconsistency in terminology that makes it hard to compare studies of statin treatment^7^. The International Society for pharmacoeconomics and Outcomes Research (ISPOR) defined *adherence* as *“the extent to which a patient acts in accordance with the prescribed interval and dose of a dosing regimen”* and *persistence* as *“the duration of time between from initiation to discontinuation of therapy”*^8^. We measured the proportion of patients that were still taking the prescribed statin at end of follow-up. This means that the ISPOR definitions of adherence and persistence are not truly applicable in our study.

Adherence to statins differs widely between studies and rates of adherence are generally higher in randomized controlled trials than observational studies. Observational studies are perceived to concur better with real-world settings^9^, although this is debated^10^.

In their meta-analysis of statin treatment, Lemestra *et al*. found adherence rates of 49% in observational studies and 90.3% in controlled trials^11^. In the EUROASPIRE IV study of a cross-sectional European ACS cohort, 85.7% were on lipid lowering medication >6 months after the event^12^. Data on adherence over longer periods are scarce, but Perreault *et al*. found in a secondary preventive cohort that adherence dropped to 71% during the first 6 months and further to 45% after 3 years^13^. The results in both our study groups were similar to the relatively high first-year adherence rates in the EUROASPIRE IV study and those of clinical trials, but high adherence was maintained during the long term. A high level of statin adherence (>80%) is associated with decreased rates of cardiovascular events, all-cause mortality and reduced health care costs^7,14,15^. This effect is further potentiated with longer treatment duration, as well as with as-low-as-possible LDL-C^2,14^.

Individual reasons for non-adherence regarding statin treatment after ACS have been poorly investigated. In meta analyses of adherence, female gender, low education, high-dose treatment, polypharmacy and poor prescriber-patient relationships have been highlighted. In other studies, the doctor’s decision to stop treatment was the most prevalent reason for treatment discontinuation, followed by side effects^16,17^. In the USAGE study, side effects were the primary reason for treatment discontinuation by 62% of patients (17%)^18^.

In this study, the single most adequate reason for treatment discontinuation was recorded prospectively. Thus, doctor’s decision to stop treatment could be part of the answer, but if additional information was available to provide a more specific cause, the more specific reason was chosen. This may explain why the leading cause of non-adherence in our intervention group was advanced disease compared to previous studies. Subjective side effects were also highly prevalent, as in previously published data^16,17^.

Reported side effects of the statin group are mainly muscle symptoms in 7–29% of patients^19^, but also included rhabdomyolysis, memory impairment, cataracts, renal dysfunction and diabetes^4^. Whether statins are subject to high prevalence of side effects or an unjust nocebo effect, placebo-controlled trials have not been able to show increased levels of side effects^3,10,20,21^. Because muscle pain is widely present in the elderly and atherosclerotic population, it is difficult to verify a true relation to treatment. We required a temporal relationship as well as a distinct regression of symptoms after treatment discontinuation to recognize a side effect compelling. Re-challenge of treatment with reappearance of symptoms would provide more definite proof of association, but many patients do not accept such provocation. Instead, we conducted a prospective review of medical records to see whether the symptoms persisted or terminated permanently. Rates of clinically judged statin-related side effects were low in both groups in our material.

About one-fifth of the study population discontinued a first time, more in the intervention group. Re-initiation is a common procedure in observational statin studies, applied to 50–75% of those that discontinue^22,23^. Unfortunately, re-discontinuation thereafter is relatively common^24^. The reasons for the first event of discontinuation in our study were predominantly avoidable.

As stated above, the proportion of non-avoidable reasons doubled for permanent discontinuation and became the most prevalent argument in the intervention group, with increased adherence as result. This finding emphasizes the need for long-term follow-up to avoid non-adherence.

In this study, there was a significant difference between men and women regarding discontinuations. In a follow-up study of USAGE to further investigate the reasons for non-adherence, previous users were more likely female, <65 years of age and had fewer cardiovascular co-morbidities then present users^25^. One suggested explanation is that women were more attentive to their health, which may lead to increased concerns about medications and their potential adverse effects^26^. Others claim that it is a result of gender inequality in the design of health care systems and thus should be seen as a modifiable risk factor^27,28^.

STEMI as including event was associated with increased adherence in this study. This has been observed previously and explained by a more frequent use of PCI, treatment at tertiary care centres and lower percentages of women in the STEMI population^29^.

Age has previously been identified as a predictor of non-adherence. Patients younger than 50 years of age and especially older than 75 years, have shown lower levels of adherence to statin treatment^30^. In the present study no association was found between age and non-adherence. However, dementia and advanced disease, factors related to older age, were common reasons for discontinuation.

In the USAGE study of a US statin-prescription database, a long and trustworthy patient-prescriber relationship was the most important factor for adherence^18^. Interestingly in the present study, the intervention group showed an initial increased tendency to discontinue treatment, but thereafter increased adherence compared with controls. Our interpretation is that individualised treatment optimization with dose and drug adjustments also increased the awareness and prescriber-patient nocebo effect. However, with continual follow-up, a joint trust and understanding of symptoms and their cause develops. Further, annual scheduled contact works as a reminder during long-term treatment and underscores its importance. Our intervention group also had a larger proportion on potent lipid-lowering medication and a significantly lower mean LDL-C at last follow-up, which indicates a more active treatment regimen. This somewhat contradicts the previous results of reduced adherence with increasing intensity of treatment, or at least that this barrier for adherence is manageable^5^. Even though medication at discharge today to a large extent meet guideline recommendations and adherence is high, the low fulfilment of the LDL-C target is probably attributable to a lack of post-discharge titration of therapy^12^.

### Strengths and limitations

This was a population-based study with minimal exclusion criteria to increase external validity. However, the randomized cohort included those patients that were capable of communicating by phone and shorter commutes, and therefore became to some extent selected. We have not found any studies with a prospective follow-up of adherence during several years in unselected cohorts. Adherence studies are generally based on registries without information regarding actual intake of the medication. Our data relied on patients’ self-reporting. A risk with this method is the possibility that patients do not report the correct information. To increase internal validity, we used a combination of interview and lipid data.

Because the study design included interviews of subjects in the non-intervention study arm, we cannot exclude that heightened awareness did not affect control subjects. However, we believe that such an effect would have had a false negative result regarding between-group differences, rather than a false positive result.

## Conclusion

A nurse-based, long-term follow-up with medical titration by telephone after an ACS resulted in higher adherence to statin treatment compared with usual care, even though a larger proportion of patients were treated with high-intensity LDL-C-lowering regimens, resulting in a lower mean LDL. A certain number of unsubstantiated discontinuations seems unavoidable, but the proportion of avoidable causes for discontinuation was reduced with duration of follow-up. Our study underscores the need for a patient-centred secondary prevention programme that extends for a longer period of time.

## Methods

### Design

Patient data were obtained from the ongoing NAILED-ACS trial, a single-centre randomised controlled trial to improve secondary prevention after ACS. The trial is nurse-led and telephone-based at a population level. The trial protocol was published previously^31^.

### Trial participants

The Östersund County Hospital is the only secondary caregiver in Jämtland County, Sweden. The catchment area is vast and rural, with a population of 130 000. All patients admitted at Östersund Hospital for ACS between 1 January 2010 and 31 December 2014 were eligible for inclusion, to enable at least 3 years of follow-up (until 31 December 2017). Exclusion criteria were limited to physical or cognitive inability to adhere to a telephone-based design (e.g., aphasia, deafness, dementia, severe disease not indicated for secondary prevention, non-Swedish/English-speaking) or participation in another clinical trial. We defined ACS according to the universal definition of myocardial infarction as acute myocardial infarction (AMI) type 1 or unstable angina (UA) with symptoms and electrocardiographic (ECG) changes indicative of ischaemia^32^. To confirm our screening routine, we performed an initial 3-month chart review which showed that all eligible patients were identified.

### Recruitment and randomization

Included patients provided written informed consent and were thereafter randomized in a 1:1 manner by a computer-generated allocation sequence in blocks of four, stratified for sex and type of ACS. Randomization resulted in an *Intervention* group followed up by a study nurse and a *Control* group with usual care follow-up by a general practitioner (GP). All patients regardless of group allocation were subject to the ordinary ACS routine with ≥2 visits (one nurse and one physician) to the cardiology out-patient clinic.

### Data collection

After inclusion, we collected baseline data through patient interview and chart review in-hospital. We recorded present medication, prevalent risk factors, prior cardiovascular history and other co-morbidities.

Baseline was defined as 1 month post-discharge. Prior to contact, blood specimens for LDL-C and blood pressure measurements performed in a standardized manner (sitting, right arm, after 5 minutes rest) were collected at the nearest health care facility and reported to the study nurses. LDL-C was calculated with the Friedwald formula from total serum cholesterol analysed with Cobas 6000 c501 (Roche, Germany). The study nurses contacted all patients at baseline to assess risk factors for secondary prevention (e.g., smoking, diet, sedentary lifestyle and medication adherence). Basis chart for the interview can be found online as Supplementary Table S1 and explanation of variables of interest translated to English in Supplementary Table S2. Thereafter, follow-up with the same routine was performed annually until the last scheduled follow-up, exclusion, patient withdrawal or death.

#### Medication adherence

We use the term *adherence* to refer to patients’ self-reported intake of prescribed treatment at each assessment. At each annual interview, patients were asked to verify ongoing medication against the current list of drugs in the medical record. Ongoing lipid-lowering medication was also related to current blood lipid values. If any uncertainty remained, a more specific discussion about ongoing medication took place. If changes in medication had been made outside the study, the new dose and drug were recorded. If treatment had been modified or discontinued, the date and reason thereof were recorded. If required, we collected additional information on adherence from the joint medical record of in-hospital and primary care. All statin discontinuation events were recorded.

We classified the following reasons for treatment discontinuation: lack of motivation, side effect with a compelling relation to medication, side effect without a compelling relation to medication, intervention by another doctor, advanced disease, dementia, misunderstanding by patient and cost related. A compelling side effect was defined as for statins reported side effects which were clearly associated with start of treatment and with a distinct improvement after discontinuation of medication. Treatment-related pathological blood tests regarding liver function [aspartate aminotransferase (ASAT), alanine aminotransferase (ALAT), alkaline phosphatase (ALP), bilirubin] or muscles (CK) were also considered compelling side effects. A non-compelling side effect did not fulfil these criteria. Advanced disease, dementia and compelling side effects were considered non-avoidable reasons for treatment discontinuation. The others were classified as potentially avoidable by the health care system or by patient- and/or doctor-related factors. We considered statin treatment a “high-intensity LDL-C lowering regime” if it had the potential to reduce LDL-C by >50% (atorvastatin 40–80 mg, rosuvastatin >10 g, simvastatin >40 mg)^2^.

### Intervention and follow-up

#### Intervention

The study nurse guided patients in a discussion of lifestyle risk factors at each interview. Depending on the outcome of blood lipid and blood pressure measurements, the nurse and a joint study physician made personalised medication adjustments to reach set targets. These adjustments were then re-assessed after 4 weeks, and further adjustments were made if needed until set targets were attained or deemed unachievable.

#### Control

At each interview the same assessments of risk factors and treatment were made, but there was no counselling in lifestyle management. We forwarded the test results of blood specimens and blood pressure to each patient’s GP. If more pressing conditions were discovered (e.g., diabetes), the patient’s GP was addressed directly.

### Outcomes

This study primarily aimed to measure adherence and specify reasons for both first and permanent discontinuation of statins in both intervention and control groups. We also aimed to identify predictors for non-adherence. Because intensity of treatment is known to correlate with side effects, we report mean LDL-C at last follow-up as well as intensity of statin therapy. As we aimed to specifically measure adherence to statin therapy, those who were only treated with another class of anti-lipid therapy (e.g., ezetimibe) were treated as non-statin users.

### Statistical analysis

We treated analyses as intent-to-treat: patients not adhering to treatment were included and those permanently lost to follow-up were excluded. We present baseline characteristics as mean for continuous variables and percentages for categorical variables. To compare the two groups, we used the two-sided independent samples t-test and chi^2^ test accordingly for continuous and categorical variables. We used Kaplan-Meier estimates to illustrate cumulative incidence of non-adherence over time for both first event and permanent discontinuation of statin treatment. To assess predictors for non-adherence, we used multivariable Cox-regression analyses of baseline characteristics with step-wise exclusion based on level of significance. Initial variables in the regressions were age, sex, education level, type of ACS at inclusion, revascularisation procedure, previous AMI or stroke/TIA and allocation at randomization. The assumption of proportional hazards was verified using scaled Schoenfeld residuals. Overall, a *p*-value <0.05 was considered significant. All statistical analyses were performed using IBM SPSS statistics software version 23.

### Ethics

This study received ethical approval from the Regional Ethics Committee, Umeå, Sweden. All data generated or analyzed during this study are included in this published article. The study was conducted in accordance with relevant guidelines and regulations. All participants signed an informed consent prior to randomisation.

### Trial registration

#### Trial number

International Standard Randomized Controlled Trial Number (ISRCTN): 96595458; http://www.controlled-trials.com/ISRCTN96595458 (Archived by WebCite at http://www.webcitation.org/6RlyhYTYK) assigned 24/08/2011. We completed this registration after the first inclusion, before the strict requirement of prospective registration of the ICMJE came to our attention. The study classifies therefor as retrospectively registered. The authors confirm that all on-going and related trials for this intervention are registered.

## Supplementary information

Supplementary Table S1: Basis chart for follow-up interview, month 1–72

Supplementary Table S2: Variable explanation for Basis chart for follow-up interview

## Data Availability

Due to legal regulations in Sweden, our complete dataset cannot be available through public repository services. All data will be supplied upon request to the corresponding author.
