# Author Correction: Statin treatment after acute coronary syndrome: Adherence and reasons for non-adherence in a randomized controlled intervention trial

**DOI:** 10.1038/s41598-021-85469-y

**Published:** 2021-03-15

**Authors:** Daniel Huber, Christian Wikén, Robin Henriksson, Lars Söderström, Thomas Mooe

**Affiliations:** 1 Department of Public Health and Clinical Medicine, Unit of Research, Education and Development - Östersund, Umeå University, Östersund, Sweden; 2 Unit of Research, Development and Education, Region Jämtland Härjedalen, Östersund Hospital, Östersund, Sweden

Correction to: *Scientific Reports* https://doi.org/10.1038/s41598-019-48540-3, published online 19 August 2019

The original version of this Article contained errors in the spelling of the authors Daniel Huber, Christian Wikén, Robin Henriksson, Lars Söderström and Thomas Mooe, which were incorrectly given as Huber Daniel, Wikén Christian, Henriksson Robin, Söderström Lars and Mooe Thomas.

This error has now been corrected in the PDF and HTML versions of the Article, and in the accompanying Supplementary Information files.

