# Supplementary material for: Statin treatment after acute coronary syndrome: Adherence and reasons for non-adherence in a randomized controlled intervention trial
Source: Sci Rep. 2019 Aug 19;9:12079. doi: 10.1038/s41598-019-48540-3 (PMC6700346; doi:10.1038/s41598-019-48540-3)
Supplement: Supplementary file 1 — Supplementary Table S1: Basis chart for follow-up interview, month 1–72 [file 41598_2019_48540_MOESM1_ESM.pdf]

NAMN P NR

Supplementary Table S1: Basis chart for follow-up interview, month 1 - 72

Statin treatment after acute coronary syndrome:

Adherence and reasons for non-adherence in a randomized controlled intervention trial

Huber Daniel, Wikén Christian, Henriksson Robin, Söderström Lars, Mooe Thomas

Samtycke Om nej - samtycke journaluppföljn.? Kommentar vb

nr	studie	fod_dat	sex	age	in_dat1	tidsy
osakerhet	orsak_sy	height	weight	bmi	waist	hip
bukhojd	whr	seiink	utbildn	ekg1	ekglys	systbt1
diastbt1	hered	hered2	smoke	cigperd	ar_rokt	trlys
tidsy_ly	tidtrlys	tiddiffl				
trop_max	tginl	kolinl	hdlinl	ldlinl	hbinl	lpkinl
tpkinl	crpinl	tidcrpin	creainl	gfrinl	fPgluinl	hba1cinl
systsit1	diassit1	syssta1	diaststa1			
				UA	NSTEMI	STEMI
CVLisch	CVLblod	TIA	CVLsida	Pares	Facialis	Syn
Dysfasi	Kognit	DTdat	DTfynd	DTstorl	Karotis	Karsida
tidlip	tidchol	tidldl	tidsysbt	tiddiabt	tidap	tidami
tidpci	tidcabg	tidstr	tidpad	tidkol	tidchf	tidht
tiddm						
dmmedink	diurink	nitroink	digink	lipink	dosstin	calcink
betaink	alfaink	brdilink	aceiink	arbink	asaink	clopink
akink	persink					
dmmedut	diurut	nitrout	digut	liput	dosstut	calcut
betaut	alfaut	brdilut	aceiut	arbut	asaut	cloput
akut	persut	rtginl	ukginl	ukgfunkt	ukghyper	

2019-06-23

NAMN P NR

ff	ffchads2	waranff	ffwkontr	ekgrytm1	ekgqrs1	ekgstt1
SokLyon1	Cornell1	LVstrain1	RomEst1	Perugia1	ut_dat1	v_tid1
tillnus1	indatnus	utdatnus	v_tidnus	tillosd	in_dat2	ut_dat2
v_tid2	v_tidtot	utdattot	interv1	int1dat	sviktinl	ischinl
datishin	dagishin	reinfinl	datreinl	dagreinl	strokinl	datstrin
dagstrin	dodinl	dodorsin	datdodin	dagdodin	endpinl	
		rankinl		malsysut	maldiaut	malsstut
maldstut	malchout	malldlut	rand_dat	interven	exklanl	utremdat

OBS LDL<1.8 vid diabetes

NAMN P NR

Löpnr Motiverad1m

rehab	indatreh	utdatreh	vtidreha	dmutreh	diuutreh	nitutreh
digutreh	liputreh	stdutreh	calutreh	betutreh	alfutreh	brdutreh
aceutreh	arbutreh	asautreh	cloutreh	akutreh	perutreh	
datum1m	CBS1m	andnod1m		sjskr1m	sjukd1m	vard1m
vard1vask	vard1dag	telssk1m	titrer1m			
smoke1m	snus1m	snussl1m	fys1m15	fys1m30	fyslat1m	fysmed1m
fysint1m	skola1m	fystran1m	strespg1m	matprg1m	rokprg1m	EQrorl1m
EQhyg1m	EQakt1m	EQsma1m	EQoro1m	Halsa1m		
dmmed1m	diur1m	sldatdiu	orssldiu		nitro1m	sldatnit
orsslnit		dig1m	sldatdig	orssldig		lip1m
dosst1m	sldatlip	orssllip		calc1m	sldatcal	orsslcal
	beta1m	sldatbet	orsslbet		alfa1m	sldatalf
orsslalf		brdil1m	sldatbrd	orsslbrd		acei1m
sldatace	orsslace		arb1m	sldatarb	orsslarb	
asa1m	sldatasa	orsslasa		clop1m	sldatclo	orsslclo
	ak1m	sldatak	orsslak		wkompl1m	pers1m
sldatper	orsslper					
chol1m	ldl1m	hdl1m	tg1m		crp1m	
syssit1m	diasit1m	syssta1m	diasta1m	waist1m		
malsys1m	maldia1m	malsst1m	maldst1m	malcho1m	malldl1m	

KOM IHÅG:
1: lägg in pat. i COSMIC för kontroll om 11 månader
2: Skriv i COSMIC t.ex.: "1 mån uppföljning enl. SPIK protokoll", ange aktuella
Ange om det framkommit något speciellt, t.ex. avvikande prover som kräver åtg

2019-06-23

3: bocka av på checklistan (Checklista TOTAL) att 1 mån besöket är gjort

NAMN P NR

Löpnr Motiverad12

datum12m	CBS12m	andnod12		sjskr12m	sjukd12m	vard12m
vard12vask	vard12dg	telssk12	titrer12			
smoke12m	snus12m	snussl12	fys12m15	fys12m30	fyslat12	fysmed12
fysint12	skola12m	fystran12	strespg12	matprg12	rokprg12	EQrorl12
EQhyg12	EQakt12	EQsma12	EQoro12	Halsa12		
dmmed12	diur12	sldatdiu12	orssldiu12		nitro12	sldatnit12
orsslnit12		dig12	sldatdig12	orssldig12		lip12
dosst12	sldatlip12	orssllip12		calc12	sldatcal12	orsslcal12
	beta12	sldatbet12	orsslbet12		alfa12	sldatalf12
orsslalf12		brdil12	sldatbrd12	orsslbrd12		acei12
sldatace12	orsslace12		arb12	sldatarb12	orsslarb12	
asa12	sldatasa12	orsslasa12		clop12	sldatclo12	orsslclo12
	ak12	sldatak12	orsslak12		wkompl12	pers12
sldatper12	orsslper12					
chol12	ldl12	hdl12	tg12	hba1c12	crp12	
syssit12	diasit12	syssta12	diasta12	waist12	weight12	
malsys12	maldia12	malsst12	maldst12	malcho12	malldl12	
ekg12	ekgrytm12	ekgqrs12	ekgstt12	SokLyon12	Cornell12	LVstr12
RomEst12	Perug12					

Värden och datum för dessa vid titrering från 1 månadskontr för interventions pa

	Systsit	Diastsit	Syststa	Diaststa	TG	chol
Datum21						
Värde						
Datum22						
Värde						
Datum23						
Värde						

NAMN P NR

Datum24
Värde
Datum25
Värde
Datum26
Värde

malsys20 maldia20 malsst20 maldst20 malcho20

1: lägg in pat. i COSMIC för kontroll om 12 månader
2: Skriv i COSMIC t.ex.: "12 mån uppföljning enl. SPIK protokoll", ange aktuella lipidvärden. Ange om det hänt något anm värt.
3: bocka av på checklistan (Checklista TOTAL): 12 mån besöket; enkäter;

NAMN				P NR		
Löpnr		Motiverad24				

datum24m	CBS24m	andnod24		sjskr24m	sjukd24m	vard24m
vard24vask	vard24dg	telssk24	titrer24			
smoke24m	snus24m	snussl24				
fys24m15	fys24m30	fyslat24	fysmed24	fysint24		
EQrorl24	EQhyg24	EQakt24	EQsma24	EQoro24	Halsa24	
dmmed24	diur24	sldatdiu24	orssldiu24		nitro24	sldatnit24
orsslnit24		dig24	sldatdig24	orssldig24		lip24
dosst24	sldatlip24	orssllip24		calc24	sldatcal24	orsslcal24
	beta24	sldatbet24	orsslbet24		alfa24	sldatalf24
orsslalf24		brdil24	sldatbrd24	orsslbrd24		acei24
sldatace24	orsslace24		arb24	sldatarb24	orsslarb24	
asa24	sldatasa24	orsslasa24		clop24	sldatclo24	orsslclo24
	ak24	sldatak24	orsslak24		wkompl24	pers24
sldatper24	orsslper24					
chol24m	ldl24m	hdl24m	tg24m	hba1c24	crp24	
syssit24	diasit24	syssta24	diasta24	waist24	weight24	
malsys24	maldia24	malsst24	maldst24	malcho24	mallдl24	
ekg24	ekgrytm24	ekgqrs24	ekgstt24	SokLyon24	Cornell24	LVstr24
RomEst24	Perug24					

Värden och datum för dessa vid titrering från 12 månadskontr för interventions p

	Systsit	Diastsit	Syststa	Diaststa	TG	chol
Datum31						
Värde						
Datum32						
Värde						
Datum33						
Värde						

NAMN P NR

Datum34
Värde
Datum35
Värde
Datum136
Värde

malsys30 maldia30 malsst30 maldst30 malcho30

1: lägg in pat. i COSMIC för kontroll om 12 månader
2: Skriv i COSMIC, t.ex.: "24 mån uppföljning enl. SPIK protokoll", ange aktuel lipidvärden. Ange om något anm värt inträffat.
3: bocka av på checklistan (Checklista TOTAL): 24 mån besöket; enkäter;

NAMN P NR

Löpnr Motiverad36

datum36m	CBS36m	andnod36		sjskr36m	sjukd36m	vard36m
vard36vask	vard36dg	telssk36	titrer36			
smoke36m	snus36m	snussl36				
fys36m15	fys36m30	fyslat36	fysmed36	fysint36		
EQrorl36	EQhyg36	EQakt36	EQsma36	EQoro36	Halsa36	
dmmed36	diur36	sldatdiu36	orssldiu36		nitro36	sldatnit36
orsslnit36		dig36	sldatdig36	orssldig36		lip36
dosst36	sldatlip36	orssllip36		calc36	sldatcal36	orsslcal36
	beta36	sldatbet36	orsslbet36		alfa36	sldatalf36
orsslalf36		brdil36	sldatbrd36	orsslbrd36		acei36
sldatace36	orsslace36		arb36	sldatarb36	orsslarb36	
asa36	sldatasa36	orsslasa36		clop36	sldatclo36	orsslclo36
	ak36	sldatak36	orsslak36		wkompl36	pers36
sldatper36	orsslper36					
chol36m	ldl36m	hdl36m	tg36m	hba1c36	crp36	
syssit36	diasit36	syssta36	diasta36	waist36	weight36	
malsys36	maldia36	malsst36	maldst36	malcho36	mallдl36	
ekg36	ekgrytm36	ekgqrs36	ekgstt36	SokLyon36	Cornell36	LVstr36
RomEst36	Perug36					

Värden och datum för dessa vid titrering från 24 månadskontr för interventions p

	Systsit	Diastsit	Syststa	Diaststa	TG	chol
Datum41						
Värde						
Datum42						
Värde						
Datum43						
Värde						

NAMN P NR

Datum44
Värde
Datum45
Värde
Datum46
Värde

malsys40 maldia40 malsst40 maldst40 malcho40

1: lägg in pat. i COSMIC för kontroll om 12 månader
2: Skriv i COSMIC, t.ex.: "36 mån uppföljning enl. SPIK protokoll", ange aktuel lipidvärden. Ange om något anm värt inträffat.
3: bocka av på checklistan (Checklista TOTAL): 36 mån besöket;

NAMN P NR

Löpnr Motiverad48

datum48m	CBS48m	andnod48		sjskr48m	sjukd48m	vard48m
vard48vask	vard48dg	telssk48	titrer48			
smoke48m	snus48m	snussl48				
fys48m15	fys48m30	fyslat48	fysmed48	fysint48		
EQrorl48	EQhyg48	EQakt48	EQsma48	EQoro48	Halsa48	
dmmed48	diur48	sldatdiu48	orssldiu48		nitro48	sldatnit48
orsslnit48		dig48	sldatdig48	orssldig48		lip48
dosst48	sldatlip48	orssllip48		calc48	sldatcal48	orsslcal48
	beta48	sldatbet48	orsslbet48		alfa48	sldatalf48
orsslalf48		brdil48	sldatbrd48	orsslbrd48		acei48
sldatace48	orsslace48		arb48	sldatarb48	orsslarb48	
asa48	sldatasa48	orsslasa48		clop48	sldatclo48	orsslclo48
	ak48	sldatak48	orsslak48		wkompl48	pers48
sldatper48	orsslper48					
chol48m	ldl48m	hdl48m	tg48m	hba1c48	crp48	
syssit48	diasit48	syssta48	diasta48	waist48	weight48	
malsys48	maldia48	malsst48	maldst48	malcho48	malldl48	
ekg48	ekgrytm48	ekgqrs48	ekgstt48	SokLyon48	Cornell48	LVstr48
RomEst48	Perug48					

Värden och datum för dessa vid titrering från 36 månadskontr för interventions p

	Systsit	Diastsit	Syststa	Diaststa	TG	chol
Datum51						
Värde						
Datum52						
Värde						
Datum53						
Värde						

NAMN P NR

Datum54
Värde
Datum55
Värde
Datum56
Värde

	malsys50	maldia50	malsst50	maldst50		malcho50

1: lägg in pat. i COSMIC för kontroll om 12 månader
2: Skriv i COSMIC, t.ex.: "48 mån uppföljning enl. SPIK protokoll", ange aktuel lipidvärden. Ange om något anm värt inträffat.
3: bocka av på checklistan (Checklista TOTAL): 48 mån besöket;

Löpnr Motiverad60

datum60m	CBS60m	andnod60		sjskr60m	sjukd60m	vard60m
vard60vask	vard60dg	telssk60	titrer60			
smoke60m	snus60m	snussl60				
fys60m15	fys60m30	fyslat60	fysmed60	fysint60		
EQrorl60	EQhyg60	EQakt60	EQsma60	EQoro60	Halsa60	
dmmed60	diur60	sldatdiu60	orssldiu60		nitro60	sldatnit60
orsslnit60		dig60	sldatdig60	orssldig60		lip60
dosst60	sldatlip60	orssllip60		calc60	sldatcal60	orsslcal60
	beta60	sldatbet60	orsslbet60		alfa60	sldatalf60
orsslalf60		brdil60	sldatbrd60	orsslbrd60		acei60
sldatace60	orsslace60		arb60	sldatarb60	orsslarb60	
asa60	sldatasa60	orsslasa60		clop60	sldatclo60	orsslclo60
	ak60	sldatak60	orsslak60		wkompl60	pers60
sldatper60	orsslper60					
chol60m	ldl60m	hdl60m	tg60m	hba1c60	crp60	
syssit60	diasit60	syssta60	diasta60	waist60	weight60	
malsys60	maldia60	malsst60	maldst60	malcho60	mallldl60	
ekg60	ekgrytm60	ekgqrs60	ekgstt60	SokLyon60	Cornell60	LVstr60

NAMN P NR

RomEst60 Perug60

Värden och datum för dessa vid titrering från 48 månadskontr för interventions p

	Systsit	Diastsit	Syststa	Diaststa	TG	chol
Datum61						
Värde						
Datum62						
Värde						
Datum63						
Värde						
Datum64						
Värde						
Datum65						
Värde						
Datum66						
Värde						
	malsys60	maldia60	malsst60	maldst60		malcho60

1: lägg in pat. i COSMIC för kontroll om 12 månader
2: Skriv i COSMIC, t.ex.: "60 mån uppföljning enl. SPIK protokoll", ange aktuel lipidvärden. Ange om något anm värt inträffat.
3: bocka av på checklistan (Checklista TOTAL): 60 mån besöket;

Löpnr Motiverad72

datum72m	CBS72m	andnod72		sjskr72m	sjukd72m	vard72m
vard72vask	vard72dg	telssk72	titrer72			
smoke72m	snus72m	snussl72				
fys72m15	fys72m30	fyslat72	fysmed72	fysint72		
EQrorl72	EQhyg72	EQakt72	EQsma72	EQoro72	Halsa72	

dmmed72	diur72	sldatdiu72	orssldiu72		nitro72	sldatnit72
orsslnit72		dig72	sldatdig72	orssldig72		lip72
dosst72	sldatlip72	orssllip72		calc72	sldatcal72	orsslcal72
	beta72	sldatbet72	orsslbet72		alfa72	sldatalf72
orsslalf72		brdil72	sldatbrd72	orsslbrd72		acei72
sldatace72	orsslace72		arb72	sldatarb72	orsslarb72	
asa72	sldatasa72	orsslasa72		clop72	sldatclo72	orsslclo72

NAMN P NR

	ak72	sldatak72	orsslak72		wkompl72	pers72
sldatper72	orsslper72					
chol72m	ldl72m	hdl72m	tg72m	hba1c72	crp72	
syssit72	diasit72	syssta72	diasta72	waist72	weight72	
malsys72	maldia72	malsst72	maldst72	malcho72	mallдl72	
ekg72	ekgrytm72	ekgqrs72	ekgstt72	SokLyon72	Cornell72	LVstr72
RomEst72	Perug72					

Värden och datum för dessa vid titrering från 60 månadskontr för interventions p

	Systsit	Diastsit	Syststa	Diaststa	TG	chol
Datum81						
Värde						
Datum82						
Värde						
Datum83						
Värde						
Datum184						
Värde						
Datum85						
Värde						
Datum86						
Värde						
	malsys172	maldia172	malsst172	maldst172		malcho172

1: lägg in pat. i COSMIC för kontroll om 12 månader
2: Skriv i COSMIC, t.ex.: "72 mån uppföljning enl. SPIK protokoll", ange aktuel lipidvärden. Ange om något anm värt inträffat.
3: bocka av på checklistan (Checklista TOTAL): 72 mån besöket;

Löpnr Motiverad84

datum84m	CBS84m	andnod84		sjskr84m	sjukd84m	vard84m
vard84vask	vard84dg	telssk84	titrer84			
smoke84m	snus84m	snussl84				
fys84m15	fys84m30	fyslat84	fysmed84	fysint84		
EQrorl84	EQhyg84	EQakt84	EQsma84	EQoro84	Halsa84	
dmmed84	diur84	sldatdiu84	orssldiu84		nitro84	sldatnit84

NAMN P NR

orsslnit84		dig84	sldatdig84	orssldig84		lip84
dosst84	sldatlip84	orssllip84		calc84	sldatcal84	orsslcal84
	beta84	sldatbet84	orsslbet84		alfa84	sldatalf84
orsslalf84		brdil84	sldatbrd84	orsslbrd84		acei84
sldatace84	orsslace84		arb84	sldatarb84	orsslarb84	
asa84	sldatasa84	orsslasa84		clop84	sldatclo84	orsslclo84
	ak84	sldatak84	orsslak84		wkompl84	pers84
sldatper84	orsslper84					
chol84m	ldl84m	hdl84m	tg84m	hba1c84	crp84	
syssit84	diasit84	syssta84	diasta84	waist84	weight84	
malsys84	maldia84	malsst84	maldst84	malcho84	malldl84	
ekg84	ekgrytm84	ekgqrs84	ekgstt84	SokLyon84	Cornell84	LVstr84
RomEst84	Perug84					

Värden och datum för dessa vid titrering från 72 månadskontr för interventions p

	Systsit	Diastsit	Syststa	Diaststa	TG	chol
Datum91						
Värde						
Datum92						
Värde						
Datum93						
Värde						
Datum194						
Värde						
Datum95						
Värde						
Datum96						
Värde						
	malsys82	maldia82	malsst82	maldst82		malcho82

1: lägg in pat. i COSMIC för kontroll om 12 månader
2: Skriv i COSMIC, t.ex.: "84 mån uppföljning enl. SPIK protokoll", ange aktuel lipidvärden. Ange om något anm värt inträffat.
3: bocka av på checklistan (Checklista TOTAL): 84 mån besöket;

NAMN P NR

Löpnr

angio1	datango1	dagango1	pci1	datpci1	dagpci1	cabg1
datcabg1	dagcabg1	ami1	datami1	dagami1		
Vid ami1 fyll även i:		NSTEMI1	STEMI1	tropm1	angina1	datang1
dagang1	svikt1	datsvik1	dagsvik1	annat1	datanna1	daganna1
stroke1	datstr1	dagstr1				

Vid stroke1 fyll även i:

CVLisch1	CVLblod1	TIA1	CVLsida1	Pares1	Facialis1	Syn1
Dysfasi1	Kognit1	DTdat1	DTfynd1	DTstorl1	Karotis1	

angio2	datango2	dagango2	pci2	datpci2	dagpci2	cabg2
datcabg2	dagcabg2	ami2	datami2	dagami2		
Vid ami2 fyll även i:		NSTEMI2	STEMI2	tropm2	angina2	datang2
dagang2	svikt2	datsvik2	dagsvik2	annat2	datanna2	daganna2
stroke2	datstr2	dagstr2				

Vid stroke2 fyll även i:

CVLisch2	CVLblod2	TIA2	CVLsida2	Pares2	Facialis2	Syn2
Dysfasi2	Kognit2	DTdat2	DTfynd2	DTstorl2	Karotis2	

angio3	datango3	dagango3	pci3	datpci3	dagpci3	cabg3
datcabg3	dagcabg3	ami3	datami3	dagami3	angina3	datang3
dagang3	svikt3	datsvik3	dagsvik3	annat3	datanna3	daganna3
stroke3	datstr3	dagstr3				

Inläggningar på sjukhus, datum (dd-mmm-yyyy):

Hjärtinfarkt from nr 4:

Hjärtsvikt from nr 4:

Stroke from nr 4:

Angina from nr 4:

Annan hjärtsjukdom from nr 4:

dodutskr	dodorsut	datdodut	dagdodut	endputsk	datendpu	dagendpu
endputyp	slut	slut_dat	slutors	dagslut		

NAMN P NR

VÅRDTID

Rött: åtgärdas direkt
Skriv ut/kopiera ekg1 !

Blått: bevakas att alla svar finns

Rött: mäts när stabilt, om möjligt nära utskrivning

Grönt: endast vid AKS
Gult: endast vid CVL

Orange: fyll i när tid finns

Gör klar enkäter för utskick, informera pat. om detta

NAMN P NR

Svart: fylls i av dr

Skriv ut svar på:

UKG
rtg pulm, DT hjärna
ultraljud halskärl,
ev. MR hjärna

rankinl gäller
utskrivningsstatus

Randomisera innan
utskrivning och fyll i
listan hos sekr.!

NAMN P NR

1 MÅNADS KONTR

Rehab vård

Rött: motsvarar
ung. SEPHIA

Grönt: endast AKS

Blått: läkemedel.
Kommentera vb
med ytterligare
uppgifter som kan
vara av betydelse

Biverkningar? -
noggrann beskrivn.!

Labsvar

Blodtryck/midja

Mål uppnått?
OBS LDL<1.8 vid dia

blodtryck och lipidvär
gärd.

NAMN P NR

1 ÅRS KONTR

Rött: motsvarar
ung. SEPHIA

Grönt: endast AKS

Blått: läkemedel.
Kommentera vb
med ytterligare
uppgifter som kan
vara av betydelse

Biverkningar? -
noggrann beskrivn.!

Labsvar

Blodtryck/midja

Mål uppnått?
OBS LDL<1.8 vid dia

Svart: fylls i av dr

OBS LDL<1.8 vid diak
LDL HDL

NAMN P NR

malldl20

a blodtryck och

NAMN P NR

2 ÅRS KONTR

Rött: motsvarar
ung. SEPHIA

Blått: läkemedel.
Kommentera vb
med ytterligare
uppgifter som kan
vara av betydelse

Biverkningar? -
noggrann beskrivn.!

Labsvar

Blodtryck/midja

Mål uppnått?
OBS LDL<1.8 vid dia

Svart: fylls i av dr

OBS LDL<1.8 vid diab
LDL HDL

NAMN P NR

mallldl30

la blodtryck och

NAMN P NR

3 ÅRS KONTR

Rött: motsvarar
ung. SEPHIA

Blått: läkemedel.
Kommentera vb
med ytterligare
uppgifter som kan
vara av betydelse

Biverkningar? -
noggrann beskrivn.!

Labsvar

Blodtryck/midja

Mål uppnått?
OBS LDL<1.8 vid dia

Svart: fylls i av dr

OBS LDL<1.8 vid diab

LDL HDL

mallldl40

la blodtryck och

NAMN P NR

4 ÅRS KONTR

Rött: motsvarar
ung. SEPHIA

Blått: läkemedel.
Kommentera vb
med ytterligare
uppgifter som kan
vara av betydelse

Biverkningar? -
noggrann beskrivn.!

Labsvar

Blodtryck/midja

Mål uppnått?
OBS LDL<1.8 vid dia

Svart: fylls i av dr

OBS LDL<1.8 vid diak
LDL HDL

NAMN P NR

malldl50

la blodtryck och

5 ÅRS KONTR

Rött: motsvarar
ung. SEPHIA

Blått: läkemedel.
Kommentera vb
med ytterligare
uppgifter som kan
vara av betydelse

Biverkningar? -
noggrann beskrivn.!

Labsvar

Blodtryck/midja

Mål uppnått?
OBS LDL<1.8 vid dia

NAMN P NR

Svart: fylls i av dr

OBS LDL<1.8 vid diak

LDL HDL

mallldl60

la blodtryck och

6 ÅRS KONTR

Rött: motsvarar
ung. SEPHIA

Blått: läkemedel.
Kommentera vb
med ytterligare
uppgifter som kan
vara av betydelse

Biverkningar? -
noggrann beskrivn.!

NAMN P NR

Labsvar

Blodtryck/midja

Mål uppnått?
OBS LDL<1.8 vid dia

Svart: fylls i av dr

OBS LDL<1.8 vid diak
LDL HDL

malldl172

la blodtryck och

7 ÅRS KONTR

Rött: motsvarar
ung. SEPHIA

Blått: läkemedel.
Kommentera vb

NAMN P NR

med ytterligare
uppgifter som kan
vara av betydelse

Biverkningar? -
noggrann beskrivn.!

Labsvar

Blodtryck/midja

Mål uppnått?
OBS LDL<1.8 vid dia

Svart: fylls i av dr

OBS LDL<1.8 vid diak
LDL HDL

malldl82

la blodtryck och

NAMN P NR

HÄNDELSER
OCH
ENDPOINTS

Första händelse=1
Andra=2
Tredje=3
